# Consumption

**Published:** 1879-08

**Authors:** E. H. Gibbs


By E. H. Gibbs, M. D.

Consumption means wasting, and when applied to disease generally refers to the lungs, although other organs of the body are subject to it. It is a wasting of the tissues from *defective nutrition.* It is not my object in this article to enter fully into a discussion of the disease, and its treatment. I will do so at another time, and this article is necessarily preliminary to such discussion.

There is and always has been a disposition on the part of invalids to jump at conclusions, and this tendency has too frequently been encouraged even by competent physicians, through carelessness, or a desire to please their patients by seeming to agree with them in opinion. Now a person may have many of the symptoms which characterize consumption, and still have no disease of the lungs whatever, and they frequently recover completely. Hence the numerous cases of miraculous recovery from a disease that never existed, and the building of fame and fortune of some easy-going doctor, who is as much astonished at his success as the patient at his recovery. Still there is no safety in such a haphazard course.

If a man is sick, ordinary prudence should dictate that he should promptly seek to know the " worst and to provide against it." Almost all diseases are curable in their early stages, hence it is of vital importance to quench the spark before it becomes a raging fire. If you have any indications of disease of the vital organs, seek promptly at any cost or inconvenience the best medical advice to be found. If this suggestion were always followed, what a world of misery it would save. There is nothing gained by closing our eyes to the inevitable, and it may cost us our life. I think, however, that every physician of large experience will agree with me in the opinion that a majority of patients who seek advice are not in so unfavorable a condition of health as they had supposed. I have space to refer to a few cases only in illustration :—

W. H. P— is a gentleman of 72 years; has had for years a slight cough every morning on getting up, and would raise a little phlegm, when the trouble would cease until the next morning. Recently these symptoms became greatly aggravated. The slight hacking in the morning changed to paroxysms of coughing many times a day, and raising of matter streaked with blood. There was fever, debility and loss of appetite, which are among the marked symptoms of lung disease. On examination I found no serious trouble with the lungs, and the spirometer showed their capacity to be undiminished. Inquiry into the history of the case, and an examination of the throat, showed that the chronic nasal catarrh, which had long existed, had extended to the bronchial tubes, in consequence of a severe cold. The unfavorable symptoms soon yielded to treatment, and in a few days he was able to attend to business as usual. In August, 1878, B. H. C——, a gentleman of sixty, called to consult me. He had supposed himself in the enjoyment of excellent health till about ten weeks previous to calling, when he began to experience loss of appetite and growing weakness. He complained of pain in the right side and in the stomach, and soon commenced to cough and to raise considerable matter and blood. He had also hectic fever and night sweats. Again I missed an opportunity that comes to some only once in a lifetime, but to me perhaps once a week, to gain credit through deception, for this patient had no lung disease, but had had inflammation of the stomach, from which he was recovering, and in a few weeks he was well. Very different from these was a case I saw about the same time. I called at the house of a friend, and there I met a young man with whom I had been acquainted some years. I saw at a glance that he was suffering from true pulmonary consumption in its last stage, and I so informed the family. They seemed quite surprised at my verdict, so quickly formed, and said that the attending physician pronounced the case typhoid fever. When the attending physician next called he was informed of my opinion and requested to call a consultation of local doctors, which was done. There were three of them, and they all agreed that the case was typhoid fever, and they expected the patient would recover. I had occasion to call again in about a week, and found the family was quite encouraged by the decision of the three doctors. I saw the sick man again, and gave it as my opinion that he could not survive thirty days, and that he better be so informed in order to give him time arrange his affairs. He died within ten days, in great agony, probably for the want of palliatives, which I had supposed all competent physicians gave in such cases. I am not acquainted with these doctors, never exchanged a word with any of them, and am not certain that I would recognize any of them were I to meet them, and consequently have no feeling toward them pro or con ; but they seem to me to belong to a class of medical men who always run in some " rut," and can't get out of it. These gentlemen all seemed to choose the same "rut"—typhoid fever. In this case, fortunately, no harm was done, for their opinion could not cause the fatal grasp of disease to loosen; but there are cases where mistakes would be fatal. I have heard of another doctor in the same neighborhood whose patients all have dyspepsia which he detects by certain peculiarities of the thumb nails. Still another doctor sees diphtheria in every throat. No matter what else they have, diphtheria is always a complication, and out come the swabs and gargles. If the disease does not exist at all, something resembling it can be developed by irritation. I will refer to only one more case in this article, and that because it possessed unusual interest. In July, last year, a medical friend called on me with one of his patients, a young man 26 years old, a clerk in a dry goods store. To the casual observer he seemed to be the picture of health; his appetite was fair, he had no cough, no pain, and not the least suspicion of lung trouble. But he complained of a constant feeling of weariness which compelled him to give up his position. I noticed that the pulse and the respiration indicated serious trouble of some vital organ, and I recalled several similar cases where the lungs were found to be the seat of the disease—particularly the case of a friend and classmate who died quite suddenly, and the cause of whose death was not suspected until a post mortem examination showed that there was hardly sufficient lung substance left to sustain life; so with this young man, the spirometer showed that there was very little healthy lung left. I saw that the case was hopeless, and so informed him. He said he felt that the trouble was serious, though he had no idea of its nature. He died six weeks later. Here again the diagnosis at this late period was not important except for the satisfaction of knowing what was doing the mischief; but there are cases numerous where life depends upon it, and no physician should venture an opinion unless he is competent to give a correct one.

